# Influence of Annealing on Polymer Optical Fiber Bragg Grating Inscription, Stability and Sensing: A Review

**DOI:** 10.3390/s23177578

**Published:** 2023-08-31

**Authors:** Hang Qu, Weiyuan Huang, Zhoupeng Lin, Xin Cheng, Rui Min, Chuanxin Teng, Christophe Caucheteur, Xuehao Hu

**Affiliations:** 1 Research Center for Advanced Optics and Photoelectronics, Department of Physics, College of Science, Shantou University, Shantou 515063, China; haqux@stu.edu.cn (H.Q.); 20wyhuang@stu.edu.cn (W.H.); 20zplin@stu.edu.cn (Z.L.); 2 Department of Electrical Engineering, Photonics Research Centre, The Hong Kong Polytechnic University, Kowloon, Hong Kong SAR 997700, China; eechengx@polyu.edu.hk; 3 Center for Cognition and Neuroergonomics, State Key Laboratory of Cognitive Neuroscience and Learning, Beijing Normal University, Zhuhai 519087, China; ruimin@bnu.edu.cn; 4 Guangxi Key Laboratory of Optoelectronic Information Processing, Guilin University of Electronic Technology, Guilin 541004, China; cxteng@guet.edu.cn; 5 Department of Electromagnetism and Telecommunication, University of Mons, Boulevard Dolez 31, 7000 Mons, Belgium; christophe.caucheteur@umons.ac.be

**Keywords:** annealing, fiber Bragg grating, polymer optical fiber, sensor

## Abstract

This article reviews recent research progress on the annealing effects on polymer optical fibers (POFs), which are of great importance for inscription, stability and sensing applications of fiber Bragg gratings (FBGs) in POFs due to their unique properties related to polymer molecular chains. In this review, the principle of annealing to reduce frozen-in stress in POFs drawing and different annealing timings are firstly summarized. Then, the annealing methods for POFs are introduced under several different conditions (temperature, humidity, strain, stress and solution). Afterwards, the principle of FBGs and several inscription techniques are reported. Subsequently, the annealing effects on the properties of POFs and polymer optical fiber Bragg gratings (POFBGs) quality are discussed. Finally, the influence of annealing on POFBG sensitivity is summarized. Overall, this paper provides a comprehensive overview of annealing techniques and their impact on both POFs and POFBGs. We hope that it will highlight the important progress made in this field.

## 1. Introduction

Similar to silica optical fibers, polymer optical fibers (POFs) have several advantages, such as small footprint, immunity to electromagnetic interference, and multiplexing capabilities [1,2]. Moreover, due to unique advantages, such as low Young’s modulus, large negative thermo-optic coefficients, high elastic strain limits, and high bending flexibility, POFs present superior characteristics for sensing applications [1,3,4,5,6,7]. Though a variety of polymer materials with specific advantages have been used for POF fabrication, such as biocompatible polymethyl methacrylate (PMMA) [8,9], cyclic olefin copolymers (TOPAS) with low water absorption [10], cyclic-olefin polymer (ZEONEX) with high glass transition temperature (T_g_) [11,12], polycarbonate (PC) with excellent clarity and engineering strength [13], and cyclic transparent amorphous fluoropolymers (CYTOP) with low losses [14], polymethyl methacrylate (PMMA) is still the most prevailing material [8]. In addition to the diversity of polymer materials, POFs can be generally classified according to the different core diameter and structure, such as step-index POFs (SI POFs) [15], microstructured POFs (mPOFs) [5,16,17], and graded-index POFs (GI POFs) [18,19,20], similar to the case of silica optical fibers.

Since the first fiber Bragg grating (FBG) was successfully inscribed in a single-mode (SM) PMMA POF in 1999 [21], FBGs have been inscribed in POFs based on TOPAS, ZEONEX, PC, and CYTOP materials either via the phase mask technique [22,23,24,25] or the femtosecond direct writing technique [26,27,28,29]. Because of POFs’ smaller Young’s modulus, larger thermo-optic coefficient and better biocompatibility compared to silica fibers, polymer optical fiber Bragg gratings (POFBGs) may be more suitable than FBGs on regular silica optical fibers for some niche sensing applications such as temperature and strain sensing, structural health monitoring and biochemical detection [30,31,32,33]. Though POFBGs have been widely inscribed in different materials and applied in different fields in recent years [1,34,35,36], the investigation on grating inscription efficiency, grating stability, sensor sensitivity and reversibility are still topics of interest in the academic community. 

The performances of POFBGs are closely related to the annealing process due to the unique polymer properties [37,38,39]. For example, in fiber drawing stage, the randomly oriented molecular chains of the polymer rearrange along the stretching direction. However, after fiber annealing at a certain temperature, the polymer molecular chains could relax and return to the original amorphous form [40,41]. Therefore, annealing process could influence the properties of polymers [42,43], and further influence the inscription, stability and application of gratings [16,37,39,44]. In addition, in PMMA-based POFs, photosensitive materials, such as trans-4-stilbenemethanol (TS) [45,46], benzyl dimethyl ketal (BDK) [47] and diphenyl disulphide (DPDS) [48,49] were doped in the fiber core to improve the grating inscription efficiency [50,51,52,53]. However, in some cases, the gratings were not stable, showing a decay performance. Fortunately, annealing after grating inscription is helpful for the regeneration of refractive index modulation and consequently promotes the recovery of the FBGs [38,54,55,56]. 

Motivated to highlight the benefits of annealing for POFBGs, we will present a comprehensive review on inscription, stability and sensing of POFBGs impacted by annealing. Considering the constant growth demand for sensor systems and the rapid development of POFBG sensors, the inscription of POFBGs and their commercial sensing applications have been proposed and summarized in previous review papers [3,57]. However, the impact of annealing on POFBGs, which is of significant importance, has not yet been reviewed. Hence, in this paper, we summarize the influence of annealing on POFBGs.

The rest of this article is structured as follows: In Section 2, the timing of annealing is reported. Section 3 introduces the conditions of POF annealing, such as temperature, strain, stress, humidity and solution. Section 4 introduces the principle of standard FBG and grating inscription technology. The annealing effect on the quality of POFBG is presented in Section 5. Section 6 focuses on the impact of annealing on the grating sensitivity including humidity sensitivity, temperature sensitivity, stress sensitivity, strain sensitivity and force sensitivity. Finally, Section 7 summarizes the main conclusions and provides an overview of the potential applications of annealing technology in the future.

## 2. Timing of Annealing

**Figure 1:**
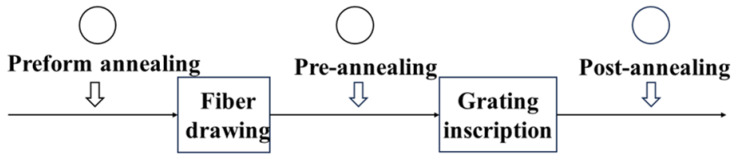
Different annealing timings including preform annealing, fiber pre-annealing and fiber post-annealing.

Annealing is a thermal treatment method that involves heating POF to a certain temperature, holding the temperature for a period of time, and then allowing the POF to slowly cool down [58]. According to the pattern of the molecular spatial arrangement, materials used for manufacturing POFs, such as PMMA, TOPAS, CYTOP, and PC, are mostly amorphous, indicating that polymer chains are randomly oriented and entangled [59]. The molecular chain of the polymer is subjected to a pulling force along the fiber axis during the process of the preform being drawn to make the POF. When the POF is extracted from the furnace and quickly cooled to room temperature to solidify, the molecular chains are immobilized, allowing the polymer molecules to align along the stretching direction [60]. The van der Waals forces are introduced between molecular chains due to their molecular arrangement, which strongly depends on the molecular orientation and could affect the thermodynamic properties of polymers [59,61], such as thermal expansion coefficient and Young’s modulus [58]. Therefore, by annealing POF at a certain temperature, because of the import of a certain amount of thermal energy, the polymer molecules are active again [40] and become randomly orientated, so that the polymer molecular chains relax and return to the original amorphous form [41], thereby eliminating the residual frozen-in stress of POFs. So far, in order to eliminate the residual frozen-in stress generated by POFs during drawing, researchers have annealed POF in three different timings, including preform annealing, fiber annealing before and after grating inscription, which are named pre-annealing and post-annealing, respectively. The timing of an annealing process is shown in Figure 1. To summarize, previous studies have shown that preform annealing before fiber drawing for a period of two weeks could improve the optical performance of POFBG and reduce the inscription time [39,61].

Pre-annealing could improve the properties of POFs, including Young’s modulus, thermal expansion coefficient as well as the photoelastic coefficient before the grating’s inscription. Meanwhile, owing to the relaxation of residual frozen-in stress, the quality of POFBG would be improved, such as reduced hysteresis, increased linear operating range and improved grating sensitivity. Pre-annealing process is typically performed in an oven or a climate chamber for a long period of heating, mostly from a few hours to a few days [15,37,39,58,62,63]. After POFBG inscription, the post-annealing process [38,64] normally takes tens of hours (most commonly around 24 h) [13,16,55,65,66,67,68], and it typically aims to reduce the hysteresis of POFBGs, adjust the Bragg wavelength, improve the stability, recover the reflectivity and improve the sensitivity of the grating.

## 3. Annealing Conditions

Annealing effect or the degree of molecular relaxation may differ in its mechanisms for polymer materials annealed under different conditions. Therefore, the annealing effect of POF depends not only on the chemical composition of the polymer material, degree of polymerization, degree of cross-linking, fiber drawing conditions, and the previous thermal history [69,70,71,72], but also on the annealing conditions [59]. In order to improve the effect of annealing on the quality of POFBG and grating sensitivity, different annealing conditions have been proposed, including temperature [69], humidity [58,66], strain, stress [73], and solution [74].

### 3.1. Temperature

Generally, the effect of annealing on POF depends on the temperature conditions of annealing. Stajanca et al. proposed that the heat supplied to the fiber at high temperatures could be viewed as an activation energy enabling molecular motions. During annealing, the degree of molecular entanglement gradually increased until the provided thermal energy was insufficient to trigger further motions or the fiber shrinkage saturation was met. Note that after annealing at a specific temperature, the fiber material structure could be generally considered stable below this annealing temperature. At higher temperatures, a considerable degree of molecular re-arrangement and fiber shrinkage may still occur [69]. Moreover, the annealing temperature applied should be higher than the β-transition temperature, in which the whole side chain of polymer molecules started to move, and the POFs began to shrink [40]. In a majority of practical cases, the most appropriate annealing temperature should be set to a point close to and smaller than the T_g_ of the polymer, since further increase in the the temperature beyond T_g_ would substantially change the whole polymer chain structure as well as the shape of the fiber [75], thus resulting in the disappearance of the inscribed grating [59]. For example, the T_g_ of PMMA, CYTOP, TOPAS 5013, ZEONEX 480R, and PC were ~105 °C [69,76], 108 °C [77], 134 °C [78], 138 °C [79], 145 °C [65], respectively. In practice, the annealing temperature was usually set at 15–20 °C below the T_g_ of the polymer.

### 3.2. Humidity

Water acts as a plasticizer during the glass transition process and with the addition of greater amounts, more significant reduction in the T_g_ of the polymer is caused. Therefore, in addition to the expected temperature dependence, the annealing process also has a strong humidity dependence. As showed in Refs. [80,81], it was found that the T_g_ of PMMA decreased to approximately 20 °C when it reached equilibrium with water, as compared to its dry state. This effect occurred due to the ability of water molecules to form hydrogen bonds with functional groups like hydroxyl and methyl on the polymer chain, leading to weakened intermolecular interactions between polymer chains and ultimately a lower T_g_ [80,82]. 

**Figure 2:**
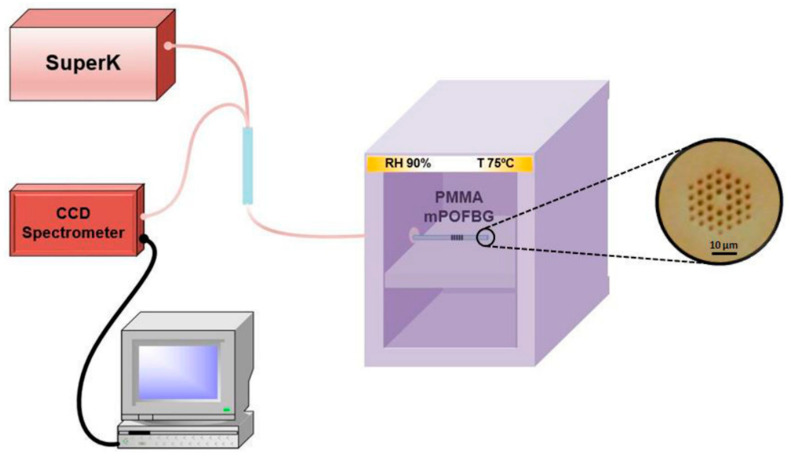
Experimental setup for PMMA mPOFBG annealing with constant humidity. Inset: Microscope image of the end facet of PMMA mPOF [66]. Reprinted with permission from [66].

**Figure 3:**
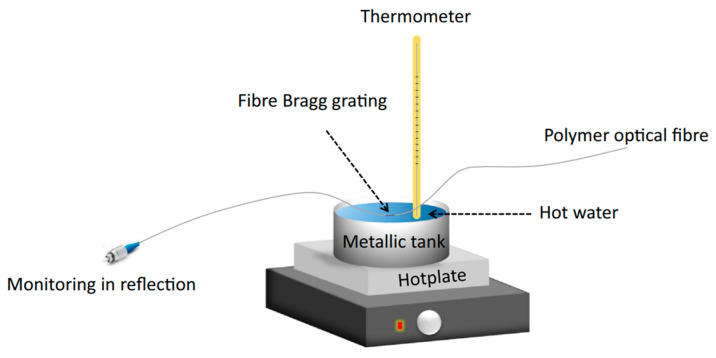
Experimental setup for POF annealing with constant humidity [59]. Reprinted with permission from [59].

Woyessa et al. reported the annealing of POF with varying relative humidity (RH). The schematic of experimental setup is shown in Figure 2. It has been demonstrated that PMMA mPOFBGs, which were post-annealed at high humidity (90% RH), exhibited superior responsivity, higher humidity sensitivity, lower hysteresis, and a higher range of working temperature than the POFBGs with or without post-annealing at low humidity [66]. Pospori et al. proposed a simple, low-cost constant humidity annealing technology. For a brief period of time, the PMMA SM mPOF was stored in a metallic tank filled with water. A hotplate was used to control the water temperature at 55 ± 2 °C and 60 ± 2 °C, as depicted in Figure 3. The hot water provided a constant 100% equilibrium RH. After annealing, the POFBG was stabilized in the environment with 40–50% RH during annealing [59].

### 3.3. Strain and Stress

Normally, POF experiences shrinkage during annealing, but if axial strain is applied during the process, the thermal expansion of the material can be restrained. As a result, POFBG is unable to freely contract during annealing. So, the strain applied during annealing should be considered.

**Figure 4:**
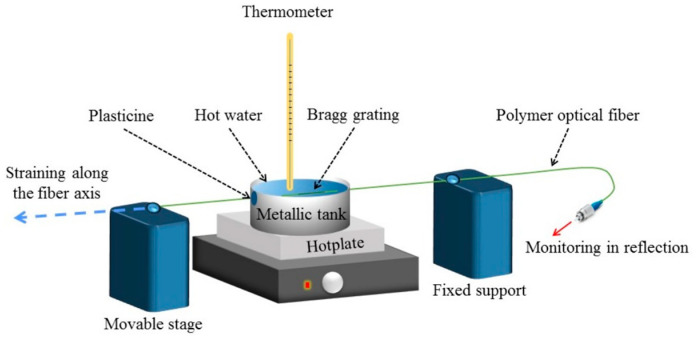
Experimental setup for POFBG annealing with constant strain [73]. Reprinted with permission from [73].

Pospori et al. applied 1% strain and 2% strain to the PMMA SM and POFBG during the post-annealing process, respectively, which could limit its thermal expansion or shrinkage in the axial direction. As shown in Figure 4, the POFBG was positioned between the high-precision movable stage and a fixed support. A constant strain was applied to the POF by moving the high-precision movable stage, which was well below the elastic limit. During strain application, the POF was placed in a metallic tank filled with water, so that the RH of the environment remained constant throughout the annealing process. Two POF apertures in the tank were sealed with plasticine to prevent water leakage. The temperature of the water inside the tank was then increased to between 20 °C and 70 °C for a short time using a hotplate underneath the tank during the annealing process. A mercury thermometer was used to monitor the temperature of water. After annealing, POF was kept at room temperature for more than one hour to release the absorbed water [73].

**Figure 5:**
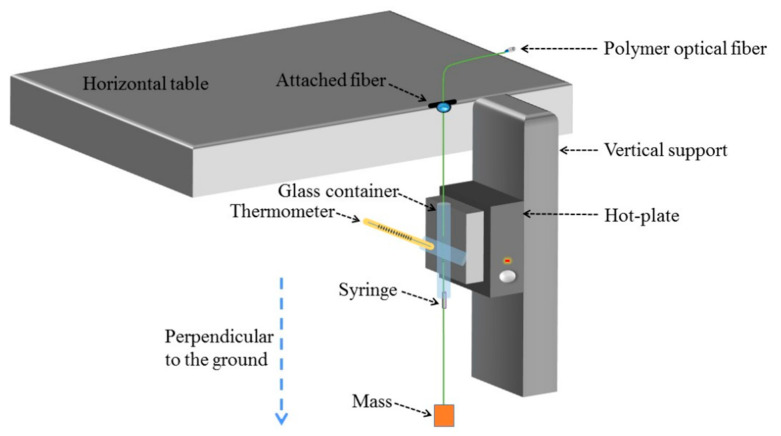
Experimental setup for the annealing process of the POFBG under constant stress [73]. Reprinted with permission from [73].

In addition to constant strain, a constant tensile force (5.8 ± 0.9 MPa and 13.4 ± 2.1 MPa) during stretching could be applied in the post-annealing process, which was also reported by Pospori et al. [73]. The experimental device is depicted in Figure 5. The POF was initially allowed to thoroughly absorb the water in the glass container for more than 40 min, before stress was imposed by increasing the mass of the blocks. The subsequent step involved annealing using a hotplate attached to the container. After the annealing process was completed, the mass was promptly removed, and the POF was kept at room temperature for a period of time to ensure stability in its Bragg wavelengths [73].

### 3.4. Chemical Treatment

Since the T_g_ of polymer materials may differ when equilibrated with chemical solutions, leading to variable degrees of residual frozen-in stress relaxation, solution-mediated annealing can be achieved without the need of a climate chamber to anneal POF. The selection of the solution primarily relies on the material of POFs and how the solution interacts with the polymers. One of the materials is methanol, which acts as the plasticizer for PMMA. According to Ref. [81], PMMA POF equilibrated in pure methanol exhibited a T_g_ that corresponded to room temperature [80,81]. Fasano et al. [74] diluted the pure methanol with water to prevent excessive relaxation of residual frozen-in stress in PMMA mPOF caused by its extremely low T_g_. Therefore, PMMA mPOFs were immersed in varying volumetric concentrations (*v*/*v*) of methanol/water solution for annealing at room temperature. Annealing PMMA mPOF equilibrated with methanol/water solution at room temperature demonstrated a similar effect to annealing POF without solution at high temperature and controlled humidity [74].

## 4. Inscription of POFBG

FBG is a kind of optical transmission device with periodic refractive index structure. FBG usually refers to the grating structure inscribed in a fiber core with grating period less than 1 μm [83], which uses the Bragg diffraction principle to achieve light splitting, filtering and other functions. Since the first successful fabrication of POFBG reported in 1999 [21,84], the inscription technique of FBGs in POFs has been studied extensively. Common methods of FBG inscription include phase mask lithography and femtosecond laser (fs) direct writing technology [22,23,24,25,26,27,28,29].

### 4.1. Fiber Bragg Gratings

**Figure 6:**
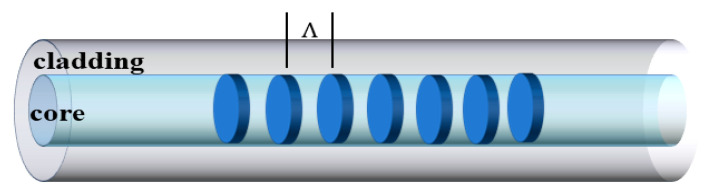
The structural diagram of standard FBG.

The structural diagram of FBG is shown in Figure 6. The Bragg condition is expressed with [85]

(1) $$\lambda_{B}=2n_{eff,core}\Lambda$$

where $n_{eff,core}$ is the effective index of the core mode, Λ represents the period of the modulation of the index along the fiber axis [83,86], and $\lambda_{B}$ represents the Bragg wavelength, which is determined by both effective index of the core mode and the period of the modulation of the index.

The light transmitted through the fiber core experiences scattering from each grating plane. When the Bragg condition is satisfied, the reflected light from each grating plane combines constructively in the opposite direction to create a back reflection peak at a central wavelength determined by the grating parameter [83,87]. On the other hand, if the Bragg condition is not satisfied, the reflected light from each succeeding plane can become increasingly out of phase and ultimately cancel each other out. The diffraction grating functions as a discriminating mirror by reflecting light in a narrow band centered on the Bragg wavelength, while allowing transmission of light at wavelengths outside the band.

### 4.2. FBG Inscription Method in POFs

One of the most effective methods for inscribing Bragg gratings in photosensitive fiber is the phase mask lithography technique [25,88,89], which employs a diffractive optical element to spatially modulate the laser writing beam. Another effective FBG inscription method is fs laser direct writing technique [84,90,91,92], which is usually achieved by focusing ultra-short pulsed laser beam through a high numerical aperture (NA) microscope objective and directly inscribing the grating structure on the fiber. 

**Figure 7:**
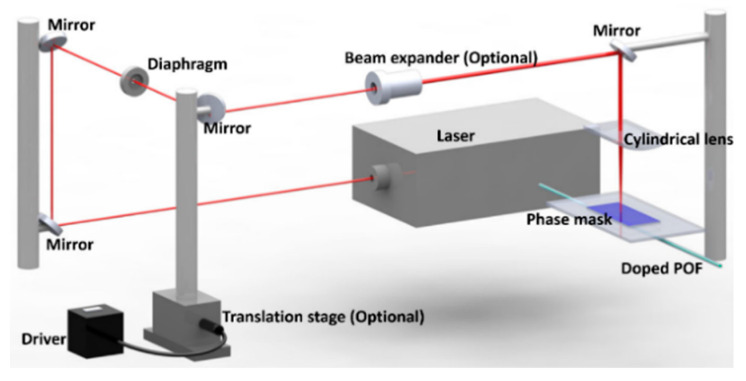
The diagram of inscription system based on laser and phase mask techniques [83]. Reprinted with permission from [83].

Both UV [89,93] and fs lasers [24,25] can be used when applying phase mask lithography to inscribe FBGs. The inscription schematic set-up is presented in Figure 7 [83]. Here, the output laser beam from the source is directed towards the POF using the mirrors [94], and the diaphragm is used to shape the laser beam. The laser beam was scanned along the fiber core via an automatically driven translation platform to increase the length of the grating. If necessary, a beam expander may also be utilized for this purpose. In order to increase power density on the fiber core, a cylindrical lens is aligned parallel to the fiber axis to focus the laser beam [89]. The POF is positioned directly behind the phase mask [89], which is used to generate beam amplitude modulation inducing refractive modulation along the fiber core [89,95].

**Figure 8:**
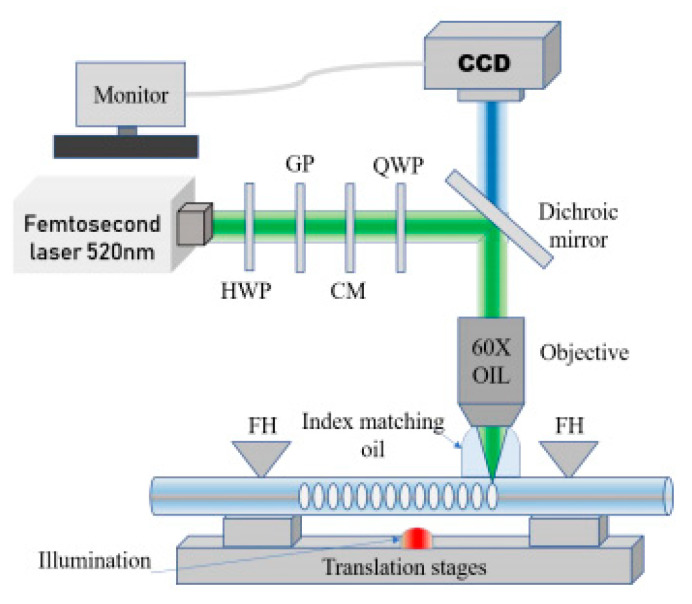
Illustration of PbP FBG inscription set-up. (HWP: half-waveplate, GP: Glan polarizer, CM: collimator, QWP: quarter waveplate, FH: fiber holder) [90]. Reprinted with permission from [90].

Femtosecond laser direct writing method for FBG inscription includes point-by-point (PbP) [58,90,96,97,98], line-by-line (LbL) [99,100,101,102] and plane-by-plane (Pl-b-Pl) techniques [103,104,105]. During the PbP inscription process, the fiber is positioned onto a high-precision translation stage [27], which moves at a constant speed along the fiber axis to generate periodic refractive index modulation induced via a focused laser beam [56,96], as shown in Figure 8 [90]. For the LbL technique, the focused fs laser beam scans perpendicular to the fiber axis. Then, the focused beam shifts with a small distance along the fiber axis for another line [99]. Similar to the LbL technique, the Pl-b-Pl technique features parallel periodic planes with a quasi-homogeneous two-dimensional refractive index change [103,104].

## 5. Annealing Influence on POFBGS

In recent years, since annealing can relax the polymer molecular chains and eliminate the residual frozen-in stress generated during fiber drawing, researchers have annealed POF under different conditions, resulting in variations in POF properties, including fiber size, thermal, mechanical, and optical properties [58,61]. In addition, the quality of the POFBG has be improved with the annealing treatment, including a reduction in grating inscription time [39], an increase in grating reflectivity [38,54,55,56], a reduction in hysteresis [62,66,68], an increase in the linear operating range [44,80], and the ability to adjust the Bragg wavelength [37,63,65,106].

### 5.1. POF Properties

For POFs, annealing can induce the relaxation of the forcibly frozen polymer molecular chains and allow them to restore their original amorphous configuration. As a result, annealing usually impacts the properties of the POFs. Sophie et al. [61] showed the impact of preform annealing on the stress-optic constant C and the constant K0. The latter is defined by the inverse Abel transform of the retardance caused by the residual birefringence of the fiber. The PMMA POF, which had a core of polyethyl methacrylate and benzyl methacrylate (PEMA/PBzMA) [107], was drawn from a non-annealed preform. The other PMMA POF, which had a core composed of PMMA doped with 2,4,6-trichlorophenyl methacrylate, was drawn from a preform that was annealed at 80 °C for 2 weeks. As a result, the PMMA POF drawn from an annealed preform had a higher stress-optic constant C and a lower constant K0 compared to the PMMA POF drawn from a non-annealed preform. 

Furthermore, Leal-Junior et al. [58] reported the effect of pre-annealing on the Young’s modulus and the thermal expansion coefficient of the PMMA POFs. They compared them to the non-annealed PMMA POF and the PMMA POFs which were pre-annealed at 70 °C under low humidity and hot water, respectively. Compared to the non-annealed PMMA POF, the pre-annealed PMMA POF exhibited a reduction in Young’s modulus and an increase in the coefficient of thermal expansion. The change in Young’s modulus may be due to the orientation relaxation of the polymer molecular structure during annealing. In addition, the Young’s modulus of PMMA POF pre-annealed under water decreased more and the coefficient of thermal expansion increased more when compared to the PMMA POF pre-annealed at low humidity, due to increased relaxation of the polymer molecular chains when annealed at higher humidity [58]. 

These studies compared the optical [61] and thermodynamic [58] properties of POF that had undergone preform annealing [61] or fiber annealing [58]. Meanwhile, the change in POF properties may cause a change in the sensitivity of the POFBG in terms of temperature, strain and so on.

### 5.2. POFBG Inscription Time

**Figure 9:**
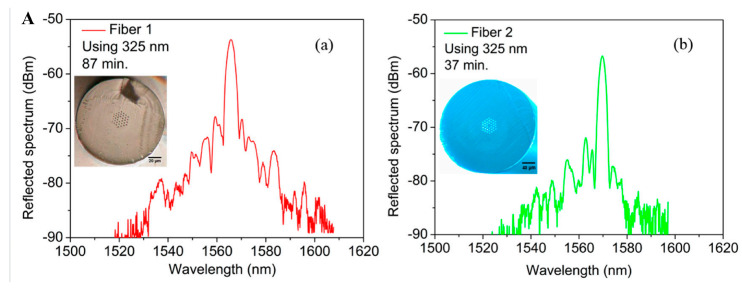

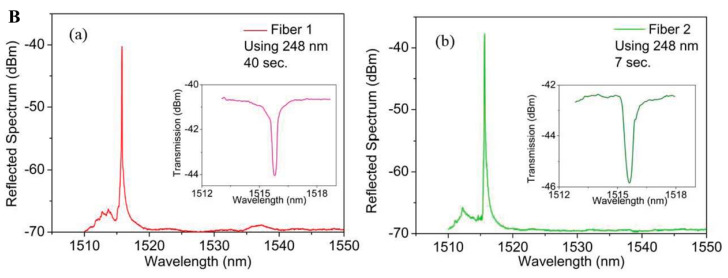
(**A**) Reflected spectra for POFBGs inscribed in (**a**) Fiber 1 and (**b**) Fiber 2 using a continuous UV He-Cd @325 nm lasers. Insets: Cross-section images of the POFs used in this work [39]. (**B**) Reflected (inset: transmission spectrum) spectra for POFBGs inscribed in (**a**) Fiber 1 and (**b**) Fiber 2 using a pulsed UV KrF @248 nm laser [39]. Reprinted with permission from [39].

In the case of POFBGs, achieving high quality POFBG requires significant refractive index modifications within a brief time. Therefore, researchers usually utilized thermal treatments to quickly inscribe POFBGs with high reflectivity [39]. Marques et al. [39] inscribed gratings in the PMMA mPOFs using two different types of UV lasers: a continuous UV He-Cd @325 nm lasers and a pulsed UV KrF @248 nm laser. Then, they demonstrated that regardless of the inscription system used to inscribe the POFBGs, the gratings inscription time with preform annealing was several times shorter than that without preform annealing, as shown in Figure 9. This might be due to the preform which contains a small quantity of water that could be extracted via annealing [39]. Since the inscription of FBGs in mPOF with the phase mask technique was a time-consuming process, preform annealing was effective in reducing the time it took to inscribe the grating.

### 5.3. Grating Reflectivity

**Figure 10:**
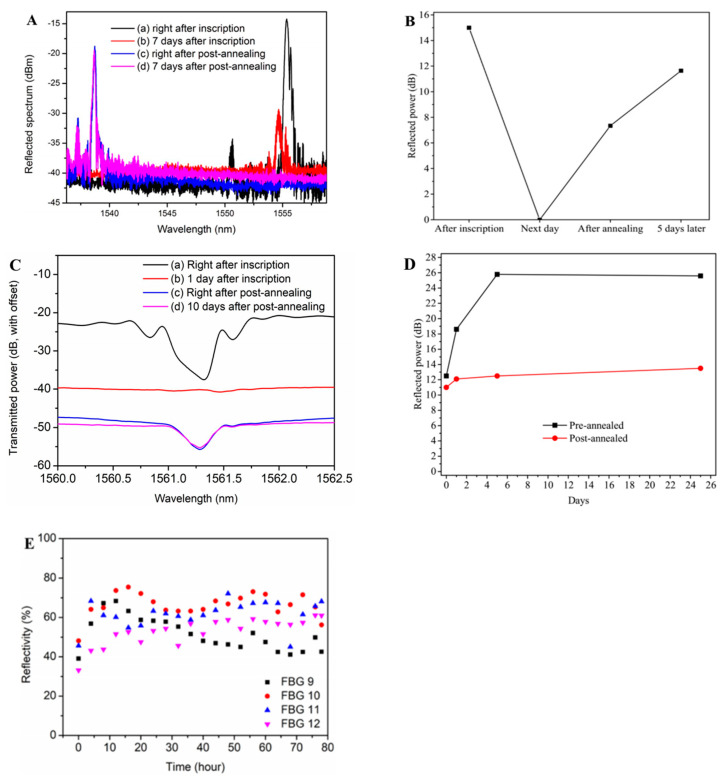
(**A**) Reflected amplitude spectra of POFBGs [(a) right after inscription, (b) 7 days after inscription, (c) right after post-annealing, and (d) 7 days after post-annealing] [38]. Reprinted with permission from [38]. (**B**) Reflected power before and after the annealing process [55]. Reprinted with permission from [55]. (**C**) Transmitted FBG spectra: [(a) right after inscription, (b) 1 day after inscription, (c) right after post-annealing, and (d) 10 days after post-annealing] [54]. Reprinted with permission from [54]. (**D**) Annealing effects on pre-annealed and post-annealed POFBGs [55]. Reprinted with permission from [55]. (**E**) The reflectivity evolutions of FBGs 9–12 during the annealing process [56]. Reprinted with permission from [56].

In some cases, the reflectivity of the grating may gradually decrease due to the inherent instability of the POFBGs. In order to restore the reflectivity of POFBGs, researchers have typically used annealing techniques [38]. Hu et al. used a He-Cd laser (Kimmon IK5751I-G) at 325 nm and the phase mask technique to inscribe FBG in a TS-doped SI PMMA POF with a highly reflectivity peak of 25 dB in 1 s, as shown in Figure 10(Aa). The grating reflectivity of the central wavelength decreased gradually during the 7 days after inscription, as shown in Figure 10(Ab). Fortunately, as shown in Figure 10(Ac,Ad), the POFBG spectrum recovered after post-annealing at 80 °C for 2 days, and also the grating reflectivity remained unchanged after 7 days. The reflectivity decay occurred because the motion of polymer chains was confined, and the recovery of the reflectivity was attributed to the fact that sufficient free volume for the movement of doped 4-stilbenemethanol molecules was generated via post-annealing [38]. Similar POFBG evolutions after inscription were presented in BDK-doped PMMA POFs by Pospori et al. [55] and Hu et al. [54], as shown in Figure 10B and Figure 10C, respectively.

Additionally, Pospori et al. fabricated two gratings to compare the grating strength evolution between pre-annealed and post-annealed POFBGs. The first one was inscribed in a pre-annealed (55 ± 1 °C for 10 min) PMMA POF with one laser pulse. The second one was inscribed in a non-annealed fiber with three laser pulses. After, the grating was post-annealed at 55 ± 1 °C for 2 min, as shown in Figure 10D, reflected power improvement over time for both gratings was obtained [55]. 

Compared to PMMA-based POFBGs, no grating reflectivity decreased in SI TOPAS/ZEONEX SM POFs inscribed with a 520 nm fs laser (SpOne-8-SHG). After inscription, the POFBGs were post-annealed at 125 °C for 78 h. As shown in Figure 10E, the average reflectivity of the post-annealed POFBGs showed a 50% enhancement of grating reflectivity compared to the POFBG without post-annealing. This increase in grating reflectivity was caused by the side-chain and backbone relaxation processes in the polymer, and the fs laser-induced grating had a long-term regenerative effect [56].

### 5.4. Hysteresis

Hysteresis of wavelength shift was observed in practical sensing applications of the high quality POFBG, especially when POFBG experienced a large strain or a high temperature. To reduce this influence, researchers annealed the POFBG before strain and temperature measurement. 

**Figure 11:**
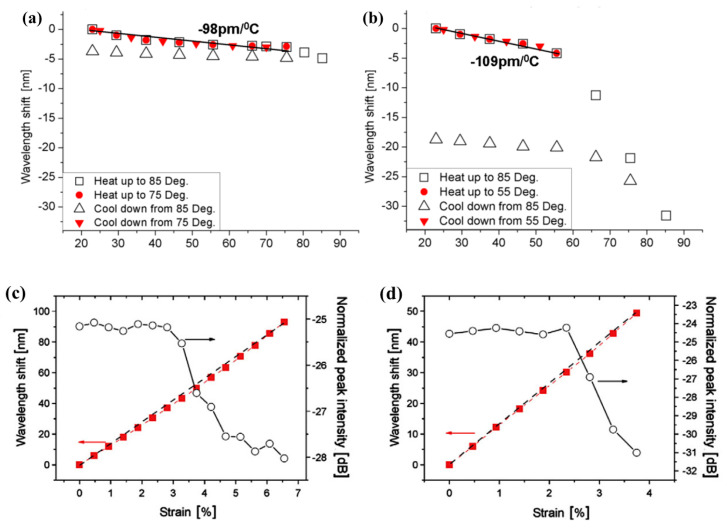
Bragg wavelength changes in pre-annealed POFBG (**a**) and non-annealed POFBG (**b**) during heating and cooling cycles. Strain tuning of pre-annealed POFBG (**c**) and non-annealed POFBG (**d**). The square-dashed line in the strain loading and unloading cycle represents the Bragg wavelength shift changes and circles-solid line represents the normalized peak intensity variation in the FBG [44]. Reprinted with permission from [44].

Yuan et al. [44] used a 325 nm continuous wave (CW) He-Cd laser (IK5751I-G, Kimmon) to inscribe FBGs in both the non-annealed and pre-annealed (80 °C for two days) PMMA SM POFs. The hysteresis of POFBG was observed once the temperature was increase to above a threshold, which was 75 °C for the pre-annealed POFBG and 55 °C for non-annealed POFBG, as shown in Figure 11a,b. The strain response of the POFBGs with mechanical stretching showed that the operational strain limits without hysteresis was 2.8% for the non-annealed POFBG and 3.8% for the pre-annealed POFBG, as shown in Figure 11c,d [44]. The result showed that the operational temperature and strain range without hysteresis of the PMMA POFBGs were extended due to pre-annealing [44]. This improvement in hysteresis in the temperature and strain responses was mostly attributable to the release of the frozen-in stress induced in the fiber drawing process via the annealing process [44]. 

**Figure 12:**
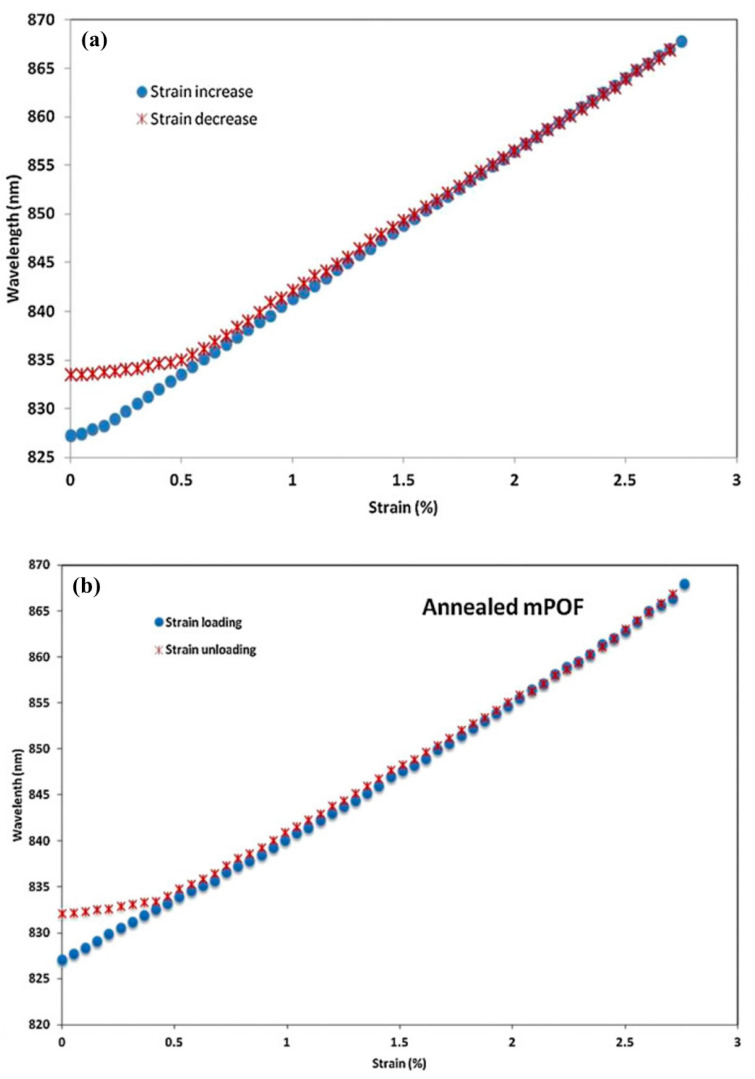
mPOF strain sensors suspended in free space showing hysteresis without annealing (**a**) and with annealing (**b**) [62]. Reprinted with permission from [62].

**Figure 13:**
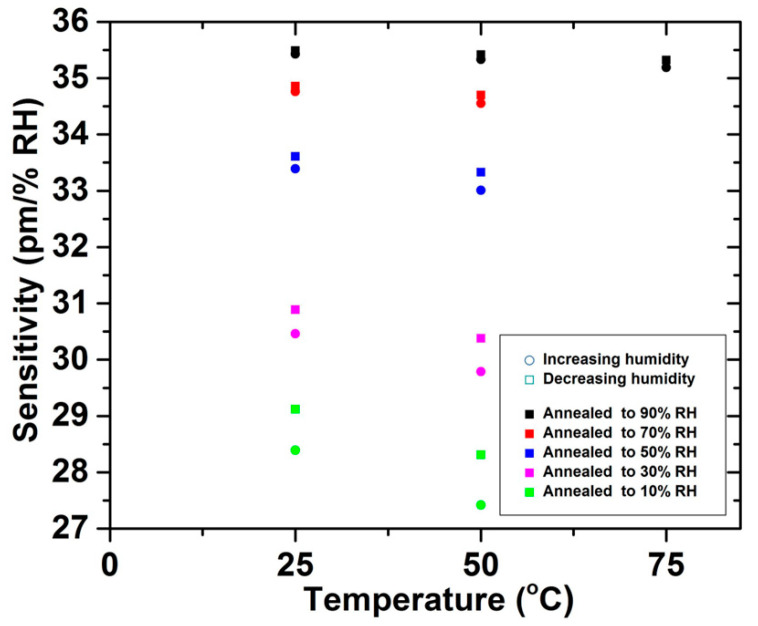
Humidity sensitivity at different temperatures for post-annealed PMMA mPOFBGs [66]. Reprinted with permission from [66].

In terms of PMMA mPOFs, Abang et al. declared that for both pre-annealed and non-annealed gratings strained to 2.75%, the hysteresis of the pre-annealed FBG was 0.34%, while the hysteresis of non-annealed PMMA mPOFBG was 0.43%, as shown in Figure 12 [62]. This phenomenon suggested that less hysteresis was for pre-annealed mPOFBG sensor, which was due to annealing leading to the relaxing of the molecules to their initial random orientation [62]. In addition, Woyessa et al. demonstrated an annealing process with both temperature and humidity control. The post-annealing processes were carried out at 80 °C for 10 h under different humidity levels, including 90%, 70%, 50%, 30% and 10% RH. Figure 13 illustrates the results of post-annealing POFBG at various humidity levels. Compared to the PMMA mPOFBGs with and without post-annealing at low humidity, the mPOFBGs post-annealed at high humidity (90%) had a superior response with a very low hysteresis, despite working at high temperature (75 °C) [66]. 

Similar conclusion was shown for the POF made of other materials. Leal-Junior et al. used a 517 nm fs for grating inscription in the CYTOP POFs. Then, they post-annealed the CYTOP POFBGs at low humidity conditions (RH ~15%) and under water (RH 100%), separately. The post-annealing temperature and time were 90 °C and 24 h, respectively. For temperature, strain and transverse force characterization, the post-annealing under water resulted in a POFBG with the lowest hysteresis when compared to the POFBGs with and without post-annealing at low humidity [68].

### 5.5. Linear Temperature Range

**Figure 14:**
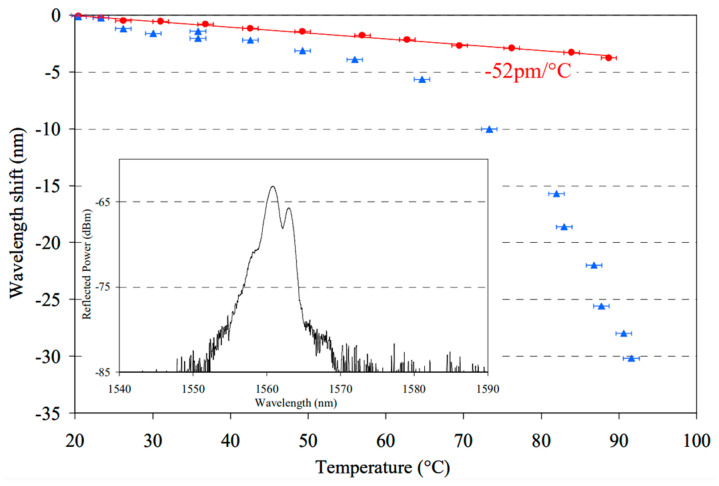
Bragg wavelength shift with temperature for pre-annealed POFBG (red) and non-annealed POFBG (blue). Inset: Grating spectrum showing double peak [37]. Reprinted with permission from [37].

For temperature measurement, the linear response of Bragg wavelength as a function of temperature cannot be maintained at a certain high temperature [37]. In order to extend the linear operating temperature range, pre-annealing could be used for POF [37]. Using a 325 nm CW He-Cd laser, Carroll [37] et al. inscribed gratings in both pre-annealed (80 °C for 7 h) and non-annealed PMMA SM mPOFs. The pre-annealed POFBG showed a linear wavelength shift with an extremely high operating linear temperature range up to 89 °C, which was higher than the non-annealed POFBG, as shown in Figure 14. This improvement is mainly because of the fact that the annealing process released the residual frozen-in stress [37].

### 5.6. Bragg Wavelength

Bragg wavelength of POFBG is also impacted by post-annealing. Heating the POF to the β-transition temperature caused the molecule chains to relax from their orientation along the fiber axis [64]. Consequently, the POF would shrink permanently, resulting in a decrease in the grating period of the POF that is proportional to the Bragg wavelength [64]. 

**Figure 15:**
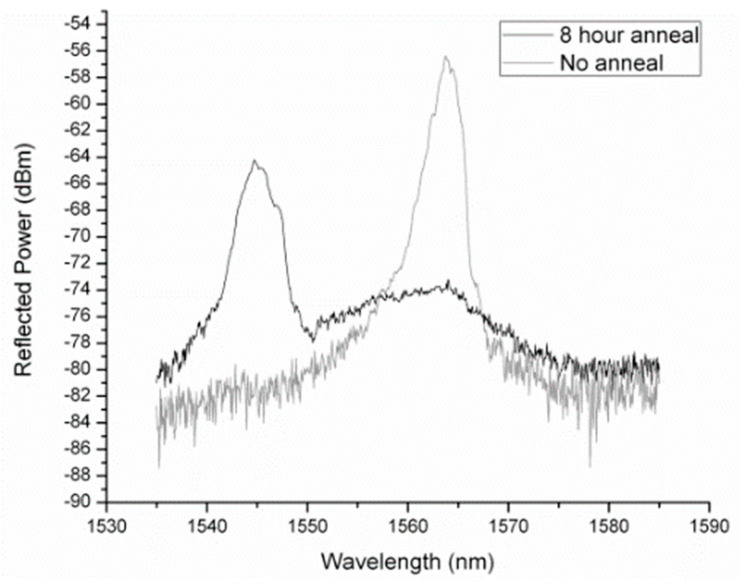
Grating spectral variation induced by thermal annealing in MM mPOF [64]. Reprinted with permission from [64].

Johnson et al. used a He-Cd laser operating at 325 nm to inscribe gratings in the PMMA MM mPOF. And then, the PMMA mPOFBG was post-annealed at 80 for 8 h. As shown in Figure 15, the Bragg wavelength shifted from the initial 1562 nm to 1545 nm [64], which was explained by the molecule chains relaxation during annealing [64]. Later, using the same technique, they created a permanent blue shift in the Bragg wavelength for manufacturing wavelength division multiplexed sensors with only one phase mask [63,106]. 

**Figure 16:**
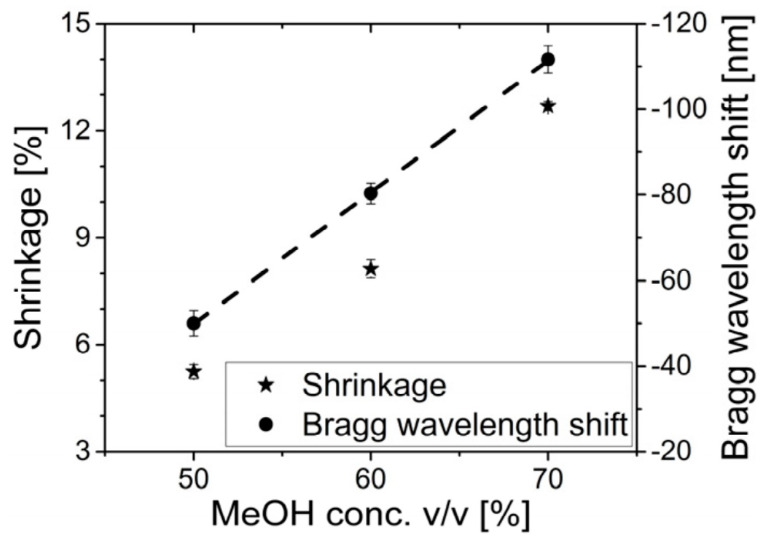
Shrinkage and Bragg wavelength shift versus MeOH concentration [74]. Reprinted with permission from [74].

To increase the blue shift, Fasano et al. proposed a solution-mediated post-annealing at room temperature. At first, they used a 325 nm CW He-Cd laser (IK5751I-G, Kimmon) and the phase mask lithography technique to inscribe gratings in PMMA mPOFs. Then, the POFBGs were post-annealed at room temperature in three different *v/v* of methanol/water solutions. The shift in the Bragg wavelength was found to be −50.0 ± 3.0 nm for 50:50% *v/v*, −80.3 ± 2.4 nm for 60:40% *v/v*, and −111.6 ± 3.2 nm for 70:30% *v/v*, as shown in Figure 16. As expected, the blue shift in the Bragg wavelength increased as the concentration of methanol was increased. This is because methanol has a stronger plasticizer effect than water, and therefore, the fiber relaxation rate increases with increasing methanol concentration [74]. 

**Figure 17:**
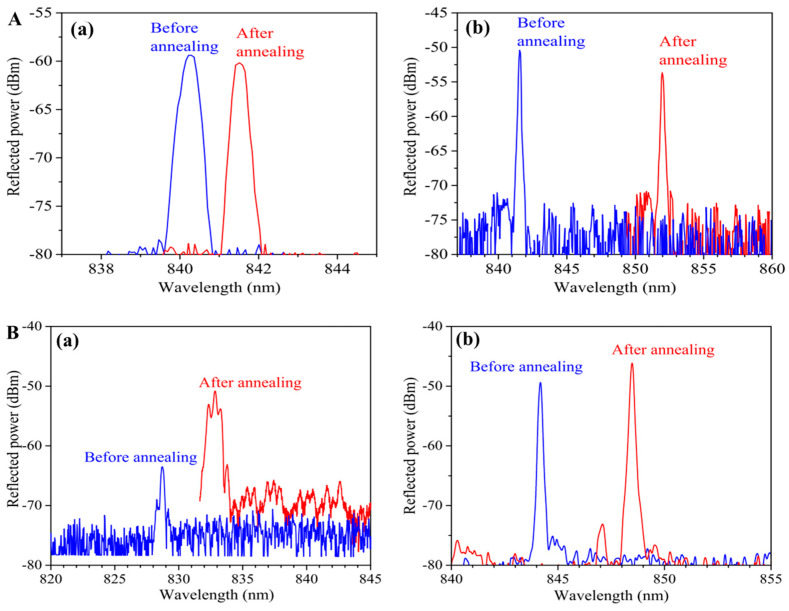
(**A**) Reflection spectra before and after annealing with constant stress of (**a**) 5.8 ± 0.9 MPa and (**b**) 13.4 ± 2.1 MPa from POFBGs inscribed with the 248 nm KrF laser [73]. (**B**) Reflection spectra before and after annealing with 1% strain for POFBG inscribed with the 325 nm He-Cd laser (**a**) and POFBG inscribed with the 248 nm KrF laser (**b**) [73]. Reprinted with permission from [73].

In addition to wavelength blue shift, wavelength red shift is also possible by changing the conditions of annealing. Pospori et al. induced the Bragg wavelength of POFBG to move to a longer wavelength by changing the conditions of annealing. They inscribed FBGs in PMMA SM mPOFs with 325 nm He-Cd and 248 nm KrF lasers. As revealed in Figure 17A, the POFBGs inscribed with the 248 nm KrF laser were subjected to post-annealing under hot water with stress levels of 5.8 ± 0.9 MPa and 13.4 ± 2.1 MPa, resulting in Bragg wavelength red shifts of 1.2 nm and 10.4 nm, respectively. Meanwhile, when POFBG inscribed with the 325 nm He-Cd laser was subjected to a post-annealing process under hot water with 1% fiber strain, the Bragg wavelength shifted from 828.7 nm to 832.8 nm, as shown in Figure 17(Ba). With the same annealing condition, the Bragg wavelength shifted from 844.2 nm to 848.5 nm for the POFBG inscribed with the 248 nm KrF laser, as shown in Figure 17(Bb). The observed redshift in Bragg wavelength was caused by the elongation of fibers attributed to thermal expansion of the polymer [73].

Since the period of the POFBG could be turned permanently longer or shorter using the post-annealing technique, it could be used to adjust POFBGs to any desirable Bragg wavelength, reducing the cost of phase masks for gratings at different Bragg wavelengths [64,73].

## 6. Annealing Influence on Grating Sensitivity

As the properties of POFs could be altered via annealing [58], the grating sensitivity could be influenced as well, such as humidity [66], temperature [15,37,39,44,63,68], stress, strain, and force sensitivity [16,39,44,59,68,108].

### 6.1. Grating Humidity Sensor

Woyessa et al. measured the humidity sensitivity of post-annealed PMMA mPOFBG at three different temperatures of 25 °C, 50 °C and 75 °C. As shown in Figure 13, compared to the mPOFBG with and without post-annealing at low humidity, the humidity sensitivity of post-annealed mPOFBG at high humidity (90% RH) was highest and even remained at a high operating temperature (75 °C) [66].

### 6.2. Grating Temperature Sensor

**Figure 18:**
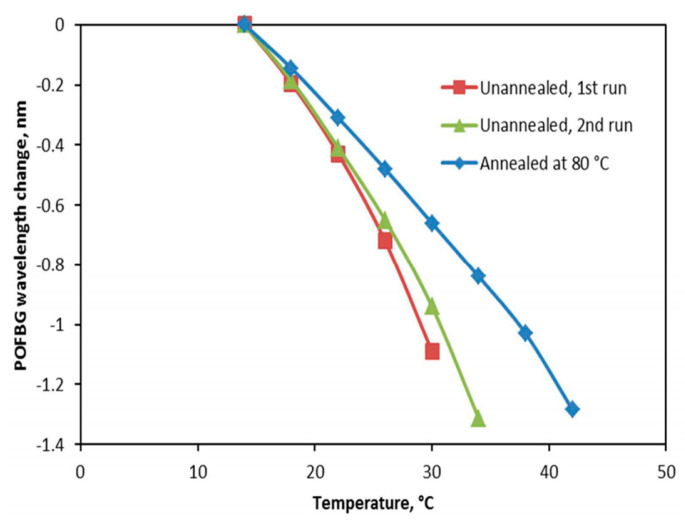
Wavelength responses comparison of non-annealed and pre-annealed POFBGs [15]. Reprinted with permission from [15].

**Figure 19:**
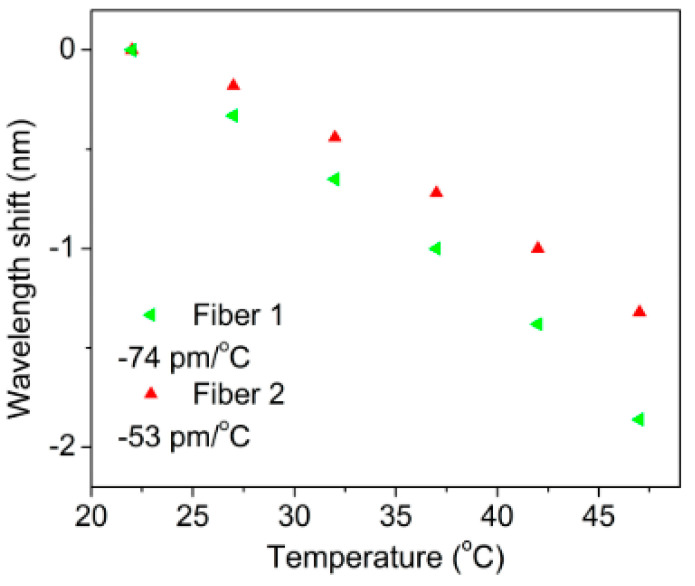
Bragg wavelength shifts in FBGs in Fiber 1 (drawn from non-annealed preform) and Fiber 2 (drawn from pre-annealed preform) at different temperatures [39]. Reprinted with permission from [39].

Zhang et al. inscribed gratings in a pre-annealed PMMA mPOF and a non-annealed PMMA mPOF using 325 nm He-Cd laser. The temperature sensitivity of the pre-annealed POFBG was found to be smaller compared to that of the non-annealed POFBG, as depicted in Figure 18 [15]. Similar effect was also demonstrated by Carroll et al. [37] and Yuan et al. [44]. Marques et al. reported the temperature sensitivity of PMMA POFBG. The one with a pre-annealed preform exhibited lower sensitivity in comparison with that of a non-annealed preform, as shown in Figure 19 [39].

As the coefficient of thermal expansion of PMMA is positive, which counteracts the contribution of thermo-optical effects to the wavelength response of POFBG [109], the temperature sensitivity of POFBG increases with decreasing coefficient of thermal expansion. Thus, the lower temperature sensitivity of annealed PMMA POFBG was because the thermal expansion coefficient of the annealed PMMA POF was larger than that of the PMMA POF without annealing [58].

Leal-Junior et al. inscribed POFBG in GI CYTOP fiber using a fs laser system (HighQ laser femtoREGEN) operating at 517 nm and using the direct writing Pl-b-Pl inscription method. The POFBGs were post-annealed at 90 °C in low humidity condition (RH of about 15%) and under water (RH of 100%) for 24 h, respectively. Finally, they found that the temperature sensitivity of POFBG post-annealed at low humidity was the highest, compared to the POFBG with and without post-annealed under water [68].

Table 1 provides a detailed overview of the POF materials, annealing conditions, and temperature sensitivity of POFBGs.

### 6.3. Grating Strain, Stress and Force Sensors

**Figure 20:**
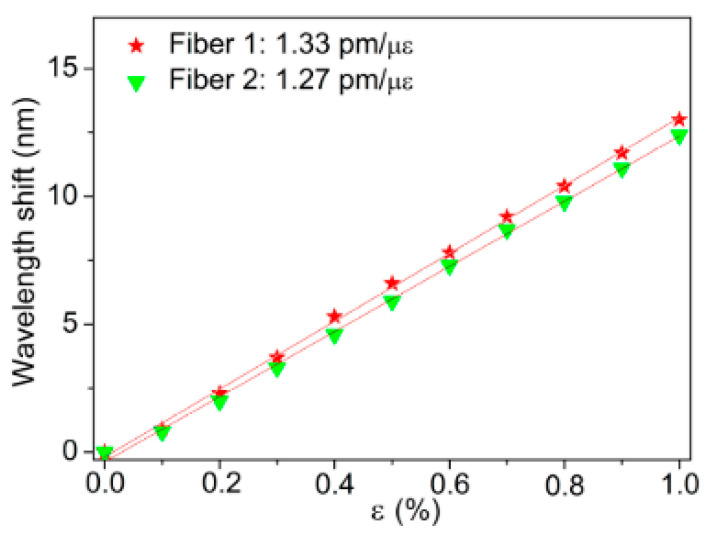
Bragg wavelength shifts in FBGs in Fiber 1 (drawn from non-annealed preform) and Fiber 2 (drawn from pre-annealed preform) under different strains [39]. Reprinted with permission from [39].

Strain, stress and force sensitivity are influenced by preform annealing, pre-annealing and post-annealing. Yuan et al. reported that the strain sensitivity of the PMMA POFBG, which was pre-annealed at 80 °C for two days before grating inscription, was higher than that for non-annealed POFBG [44]. Pospori et al. [16,59] and Marques et al. [108] discovered that post-annealed PMMA POFBGs under hot water exhibited bigger strain, stress, and force sensitivity. Marques et al. observed that preform annealing induced a different effect on the strain sensitivity of PMMA POFBG. As shown in Figure 20, the POFBG with a pre-annealed preform had a lower strain sensitivity compared to that with a non-annealed preform. This effect was possibly due to the fact that th epreform annealing process could prevent moisture diffusion [39].

Compared to the CYTOP POFBGs with and without post-annealing at low humidity, Leal-Junior et al. also showed that the post-annealed one under water had the highest sensitivity for strain and transverse force characterization [68]. Table 2 presents detailed information on the POF materials, annealing conditions, strain, stress, and force sensitivity of POFBGs.

## 7. Conclusions

This article reviews the latest progress in the application of thermal annealing techniques on POFBGs in recent years. Firstly, we described the principle of reducing the freezing stress in POFs during the stretching process through thermal annealing, and then summarized the different annealing timings of POFs, such as preform annealing, pre-annealing and post-annealing. Secondly, in order to improve the annealing technique, the emphasis was placed on changing the annealing conditions, including temperature, humidity, strain, stress, and solution type. Thirdly, the principle of standard FBG and grating inscription technology were introduced. Fourthly, the influence of annealing on the quality of gratings inscribed in POFs is discussed. Finally, the impact of annealing on the sensitivity of POFBGs is presented, including humidity, temperature, strain, stress, and force sensitivity.

Nevertheless, there are still many aspects of annealing that must be further developed for the realization of sensors in everyday life in terms of sensor usability and opportunities for application beyond laboratory-scale applications. Currently, the bottlenecks for the POFBG sensor are reliability, stability and wide measurement range. Although annealing can improve the quality of POFBGs, the long-term grating stability is still unknown. Though hysteresis can be mitigated, the effect cannot be solved totally using the annealing methods. For further improvement, the annealing conditions need to be optimized according to the POF materials. In addition, the POF fabrication process including preform fabrication and fiber drawing need to be identical to obtain quite similar inherent properties of POFs. Both of them are beneficial to the realization of large-scale POFBG sensing applications in the industry. Also, further investigation of the influence of annealing on grating stability and hysteresis reduction should be conducted for improve the grating performance. We believe that consideration of these challenges will further develop the annealing techniques and provide great opportunities for practical applications of POFBG sensors.

## Figures and Tables

**Table 1:** POF materials, annealing conditions, and temperature sensitivity of POFBGs.

Fiber Type	Fiber Material	Laser	Annealing Type	Annealing Temperature	Annealing Time	Temperature Sensitivity	Reference
SM mPOF	PMMA	325 nm He-Cd laser	pre-annealed	80 °C	7 h	−52 pm/°C	[37]
SM POF	PMMA	325 nm He-Cd laser	non-annealed	-	-	−109 pm/°C	[44]
			pre-annealed	80 °C	2 days	−98 pm/°C	
mPOF	PMMA	325 nm He-Cd laser	without annealing preform	-	-	−74 ± 2.2 pm/°C	[39]
			annealing preform	80 °C	2 weeks	−53.1 ± 3.3 pm/°C	
GI MM POF	CYTOP	517 nm fs laser system (HighQ laser femtoREGEN)	without post-annealing	-	-	19.75 pm/°C	[68]
			post-annealed at low humidity	90 °C	24 h	20.95 pm/°C	
			post-annealed under water	90 °C	24 h	5.05 pm/°C	

**Table 2:** POF materials, annealing conditions, strain, stress, and force sensitivity of POFBGs.

Fiber Type	Fiber Material	Laser	Annealing Type	Annealing Temperature	Annealing Time	Strain Sensitivity	Stress Sensitivity	**Force Sensitivity**	**Reference**
SM POF	PMMA	325 nm He-Cd laser	non-annealed	-	-	1.3 pm/με	-	-	[44]
			pre-annealed	80 °C	2 days	1.37 pm/με	-	-	
SM mPOF	PMMA	325 nm CW He-Cd laser	without post-annealing	-	-	0.664 pm/με	0.137 pm/kPa	0.0109 pm/μN	[16]
			post-annealed under water	60 ± 2 °C	2 min	0.726 pm/με	0.217 pm/kPa	0.0137 pm/μN	
			without post-annealing	-	-	0.536 pm/με	0.147 pm/kPa	0.0109 pm/μN	
			post-annealed under water	60 ± 2 °C	4 min	0.668 pm/με	0.201 pm/kPa	0.0137 pm/μN	
			without post-annealing	-	-	0.772 pm/με	0.173 pm/kPa	0.0134 pm/μN	
			post-annealed under water	60 ± 2 °C	30 min	0.944 pm/με	0.202 pm/kPa	0.0146 pm/μN	
			without post-annealing	-	-	0.714 pm/με	0.184 pm/kPa	0.0136 pm/μN	
			post-annealed under water	55 ± 2 °C	30 min	0.880 pm/με	0.220 pm/kPa	0.0165 pm/μN	
mPOF	PMMA doped with BDK	325 nm CW He-Cd laser	without post-annealing	-	-	0.68 ± 0.01 pm/με	0.14 ± 0.02 pm/kPa	0.109 ± 0.001 pm/μN	[108]
			post-annealed under water	64 ± 2 °C	2 min	0.73 ± 0.02 pm/με	0.22 ± 0.02 pm/kPa	0.137 ± 0.001 pm/μN	
mPOF	PMMA	325 nm CW He-Cd laser	without annealing preform	-	-	1.27 ± 0.01 pm/με	-	-	[39]
			annealing preform	80 °C	2 week	1.33 ± 0.01 pm/με	-	-	
mPOF	PMMA	325 nm He-Cd laser	without post-annealing	-	-	0.681 ± 0.009 pm/με	0.141 ± 0.018 pm/kPa	10.92 ± 1.37 pm/mN	[59]
			post-annealed under water	60 ± 2 °C	2 min	0.739 ± 0.019 pm/με	0.217 ± 0.022 pm/kPa	13.65 ± 1.37 pm/mN	
			without post-annealing	-	-	0.708 ± 0.007 pm/με	0.180 ± 0.023 pm/kPa	10.92 ± 1.37 pm/mN	
			post-annealed under water	60 ± 2 °C	4 min	0.902 ± 0.016 pm/με	0.260 ± 0.025 pm/kPa	14.33 ± 1.37 pm/mN	
			without post-annealing	-	-	0.771 ± 0.011 pm/με	0.173 ± 0.024 pm/kPa	13.41 ± 1.85 pm/mN	
			post-annealed under water	60 ± 2 °C	30 min	0.943 ± 0.011 pm/με	0.202 ± 0.026 pm/kPa	14.64 ± 1.85 pm/mN	
			without post-annealing	-	-	0.711 ± 0.007 pm/με	0.163 ± 0.026 pm/kPa	12.24 ± 1.96 pm/mN	
			post-annealed under water	55 ± 2 °C	30 min	0.879 ± 0.020 pm/με	0.191 ± 0.018 pm/kPa	14.37 ± 1.34 pm/mN	
GI MM POF	CYTOP	517 nm fs laser system (HighQ laser femtoREGEN)	without post-annealing	-	-	1.13 pm/με	-	−188.3 pm/N	[68]
			post-annealed at low humidity	90 °C	24 h	0.95 pm/με	-	−225.0 pm/N	
			post-annealed under water	90 °C	24 h	1.69 pm/με	-	−341.7 pm/N	

## Data Availability

No new data were created in this work.
